# A 10-year-long ongoing complete response to vismodegib as first-line treatment in adult metastatic medulloblastoma

**DOI:** 10.2340/1651-226X.2026.45543

**Published:** 2026-05-11

**Authors:** Suweydo Abdi, Paula Poikonen-Saksela, Päivi Halonen, Olli Tynninen, David A. Reardon, Micaela Hernberg

**Affiliations:** a Comprehensive Cancer Center, Helsinki University Hospital and University of Helsinki, Helsinki, Finland; b Department of Pathology, Helsinki University Hospital and University of Helsinki, Helsinki, Finland; c Center for Neuro-Oncology, Dana-Farber Cancer Institute and Harvard Medical School, Boston, MA, USA

**Keywords:** Medulloblastoma, vismodegib, hedgehog inhibitor, PTCH1 gene mutation

## Introduction

Medulloblastoma (MB) is an aggressive malignant tumor that originates from the cerebellum [1]. It is highly invasive and known to be the most prevalent pediatric brain tumor. MB is extremely rare in adults and nearly 70% of MB cases appear before the age of 10 [1, 2]. Main risk factors for MB are hereditary cancer predisposition syndromes [3].

MB is divided into four molecular subgroups: sonic hedgehog (SHH), wingless (WNT), group 3 and group 4. SHH is the second most common subgroup accounting for approximately 30% of all MB cases. The tumor originates from the cerebellar hemispheres [3]. SHH-activated TP53-wildtype MB is the most common subgroup in adults [4], and the 5-year overall survival (OS) rate of adult MB is reported to be 76% [5].

Current treatment options of MB in adults are mainly derived from pediatric protocols and consist of maximal safe resection, craniospinal radiation therapy (CSRT) and if needed, chemotherapy depending on clinical risk stratification [4]. Advances in molecular biology of MB have enabled vismodegib use in SHH-MB. Vismodegib, a synthetic small-molecule cyclopamine-competitive antagonist of the transmembrane protein smoothened homologue (SMO) receptor, inhibits the SHH signaling pathway [6]. Normally, the SHH-protein binds to the inhibitory receptor PTCH1 leading to the activation of SMO and consequently the downstream SHH signaling pathway [7]. Vismodegib was approved by the US Food and Drug Administration in 2012 and the European Medicines Agency in 2013 for the treatment of basal cell carcinoma based on objective response rate (ORR) [8]. Vismodegib has also been studied in the treatment of acute myeloid leukemia [9], acute lymphoblastic leukemia [10] and MB [11].

Herein, we report an ongoing 10-year disease control with vismodegib in the upfront treatment of an adult with metastatic recurrent SHH-MB.

## Case report

**Figure 1:**
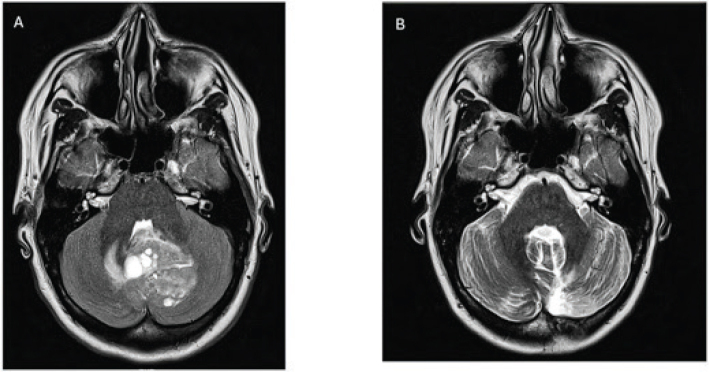
Brain magnetic resonance images. (A) In October 2013, preoperative MRI reveals a discreate mass in the cerebellum. (B) In February 2015, 16-month postoperative MRI scan shows the total resection of the tumor. MRI: magnetic resonance imaging.

In 2013, a 25-year-old Finnish woman was diagnosed with SHH-activated MB. Three to four years prior to the diagnosis, she had experienced intermittent migraine headaches with visual aura. In October 2013, she was referred to the emergency department because of a 6-week history of daily progressing headaches associated with tight neck and shoulder muscles. Brain computer tomography (CT) and magnetic resonance imaging (MRI) revealed a 4.5-cm-diameter cystic, multi-compartment well-defined tumor in the vermis and left cerebellar hemisphere blocking the fourth ventricle and causing hydrocephalus (Figure 1A). Staging evaluation included a negative spine MRI. Gross total resection (GTR) of the tumor was conducted, and histopathological examination showed desmoplastic nodular MB grade 4 with a Ki-67 proliferation index of 70–80%. Neither molecular subtyping of the tumor nor cerebrospinal fluid (CSF) cytology was done at initial diagnosis.

Postoperatively, she received craniospinal radiotherapy (36 Gy, 1.8 Gy per fraction) with a posterior fossa boost (54 Gy, 1.8 Gy per fraction). Follow-up brain and spine MRI scans 3, 7 and 10 months post-radiotherapy revealed no evidence of tumor recurrence (Figure 1B).

**Figure 2:**
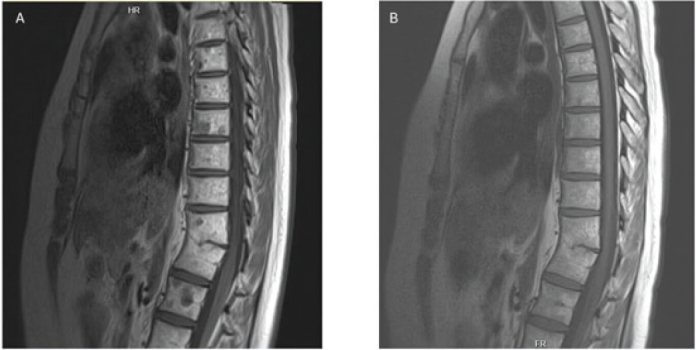
Spine magnetic resonance images. (A) In February 2015, MRI at detection of bone metastases with clear focal changes and (B) in October 2016, after 1.5 years of vismodegib therapy with no signs of lesion in corpus vertebrates. MRI: magnetic resonance imaging.

Approximately 13 months after radiotherapy, she developed pressure and tightness in the lower back. Brain and spine MRI were positive for new focal changes in corpus vertebrates indicating bone metastases (Figure 2A). A full-body CT scan revealed no evidence of additional sites of potential tumor recurrence. A bone biopsy revealed evidence of MB metastatic disease. The molecular subtype of the tumor studied by OncoPanel assay [12] revealed two mutations in the *PTCH1* gene (c.1172_1174AAG>G (p.K391fs) and c.1347_splice (p.M449_splice)) compatible with SHH-activated and TP53-wildtype MB. Following a detailed discussion of the therapeutic rationale, potential benefits and risks and the limited treatment data [13, 14], she was started on vismodegib at a dose of 150 mg orally once a day as first-line treatment. Overall, she tolerated it well apart from loss of taste, dry mouth as well as intermittent muscular cramping and morning nausea. A spine MRI conducted 2 months following the start of vismodegib showed more sclerotic and well-defined bone metastases indicating an initial response to vismodegib (Figure 2B).

Based on a theoretical rationale that alternating daily vismodegib with cycles of established cytotoxic therapy could enhance therapeutic efficacy and lessen the risk of acquired resistance, vismodegib was interrupted after 2 months and she received a course of cisplatin, cyclophosphamide and vincristine followed by filgrastim. However, the chemotherapy was discontinued after 1 month due to poor tolerance including significant nocturnal diaphoresis, hearing loss, tinnitus, fever, nausea and cytopenias. Vismodegib was restarted at a dose of 150 mg once daily.

Five months post-chemotherapy, a full-body fluorode-oxyglucose positron emission tomography-CT (FDG-PET-CT) scan revealed increased FDG-intake and active lytic foci in the left occipital bone above the former craniotomy opening. Vismodegib was paused and an occipital bone lesion was surgically removed with pathology revealing reactive inflammation and no evidence of MB. After surgery, she continued vismodegib 150 mg once daily in 1-month cycles, with 1 month of rest between cycles. She tolerated this well with a reduction in muscular cramping. The period of rest was extended from 1 month to 6 weeks and eventually to 2 months because of the stability of her cancer. Regular body FDG-PET-CT scans conducted since April 2016 have revealed no sign of abnormal tracer accumulation, consistent with ongoing remission. She electively discontinued vismodegib after 6 years of dosing and currently remains in remission 5 years after vismodegib discontinuation.

## Discussion

We present a case of a female patient with metastatic recurrent SHH-MB who has shown the longest response to vismodegib reported to date by being in complete remission for 10 years including being off therapy for the past 5 years. Besides our case report of vismodegib use as first-line in a metastatic MB, there are only four case reports of patients who received vismodegib as first-line therapy [7, 13, 15, 16]. These case reports show promising results of vismodegib treatment as initial therapy for unresectable primary tumors as well as first-line in metastatic MB patients. Vismodegib responses have been reported for both the primary tumor [16] and bone metastases [7, 13, 14, 15].

The two longest responses among these cases were 29 months and 15 months. The first case is a 51-year-old man with an unresectable undefined molecular subgroup of MB who had 29 months of response to vismodegib [16].The second case is a 24-year-old woman with an unresectable metastatic SHH-activated TP53-wildtype MB who had 15 months of response to vismodegib [7]; however, further response and follow-up data were not provided.

Li et al. published a systemic review and meta-analysis among Phase I and II clinical trials involving MB patients treated with SMO antagonists. In their meta-analysis, the pooled ORR of both SMO inhibitors, sonidegib and vismodegib, was 37% for SHH-MB but zero for other MB subtypes. The pooled ORR of sonidegib was 55% among SHH-MB, whereas vismodegib produced a 17% ORR in the same population. Sonidegib produced an ORR 1.87-fold higher than that of vismodegib in the pediatric population but demonstrated similar efficacy to vismodegib in the adult population [11].

A study by Frappaz et al. reported results of a phase I/II clinical trial evaluating vismodegib + temozolomide (TMZ) in SHH recurrent/refractory adult MB. Twenty-four TMZ-naive patients were randomized 2:1 to receive vismodegib + TMZ (arm A) or TMZ (arm B) with a plan to explore the 6-month progression-free survival (PFS-6) [17]. If PSF-6 was ≥ 3/9 in arm A at the end of the first part of phase II, patients were to proceed to the second part of phase II where the treatment would have been considered effective if PFS-6 was achieved in at least 12/25 patients. However, at the end of part one, the PFS-6 of arm A was 20% (2/10 patients), and the study was terminated.

Based on a review of clinicaltrials.gov, only one clinical trial evaluating vismodegib for MB is active but closed to recruitment. This study is focused on newly diagnosed patients (NCT01878617). Four other clinical trials on the use of vismodegib in MB on clinicaltrials.gov were either terminated [17] or completed [18] (NCT00822458). The reported study [18] was a completed phase II clinical trial where vismodegib showed modest activity against adult recurrent SHH-MB, as three adult patients with SHH-MB out of 31 showed an 8-week radiologic response. Results of another clinical trial have not been published yet (NCT00822458). No clinical trials are listed for sonidegib in the treatment of MB.

## Conclusions

Our report highlights the potential benefit of vismodegib as first-line treatment for adults with newly diagnosed metastatic and recurrent SHH-MB. In addition, vismodegib was well tolerated and the durability of disease control achieved in our case with vismodegib therapy is exceptional.

Our report also highlights the important role of molecular and genomic screening of MB to guide optimal consideration of therapy options [18]. It supports the conduct of a registration clinical trial of vismodegib as first-line in SHH-MB particularly given the modest efficacy of current standard treatment. Targeted treatment such as vismodegib may also allow delay of exposure to both short- and long-term potential side effects of cytotoxic radiation therapy and chemotherapy. Finally, considering the rarity of MB in adults, future studies will need to be conducted via international collaboration.

## Data Availability

The data supporting the findings of this case report are not publicly available due to patient privacy and data protection regulations.
